# Vaginal microbicides for reducing the risk of sexual acquisition of HIV infection in women: systematic review and meta-analysis

**DOI:** 10.1186/1471-2334-12-289

**Published:** 2012-11-06

**Authors:** Jael Obiero, Peter G Mwethera, Gregory D Hussey, Charles S Wiysonge

**Affiliations:** 1 Department of Reproductive Health and Biology, Institute of Primate Research, Karen Road, Nairobi, Kenya; 2 Department of Medical Microbiology, University of Nairobi, Off Ngong Road, Nairobi, Kenya; 3 Vaccines for Africa Initiative, Institute of Infectious Disease and Molecular Medicine, University of Cape Town, Observatory, Cape Town, South Africa; 4 Division of Medical Microbiology, Department of Clinical Laboratory Sciences, University of Cape Town, Observatory, Cape Town, South Africa

## Abstract

**Background:**

Each year more than two million people are newly infected with HIV worldwide, a majority of them through unprotected vaginal sex. More than half of new infections in adults occur in women. Male condoms and male circumcision reduce the risk of HIV acquisition; but the uptake of these methods is out of the control of women. We therefore aimed to determine the effectiveness of vaginal microbicides (a potential female-controlled method) for prevention of sexual acquisition of HIV in women.

**Methods:**

We conducted a comprehensive search of peer-reviewed and grey literature for publications of randomised controlled trials available by September 2012. We screened search outputs, selected studies, assessed risk of bias, and extracted data in duplicate; resolving differences by discussion and consensus.

**Results:**

We identified 13 eligible trials that compared vaginal microbicides to placebo. These studies enrolled 35,905 sexually active HIV-negative women between 1996 and 2011; in Benin, Cameroon, Cote d’Ivoire, Ghana, Kenya, Malawi, Nigeria, South Africa, Tanzania, Uganda, Zambia, Zimbabwe, India, Thailand, and the United States of America. A small trial of 889 women found that tenofovir (a nucleotide reverse transcriptase inhibitor) significantly reduces the risk of HIV acquisition (risk ratio [RR] 0.63, 95% confidence intervals [CI] 0.43 to 0.93). Effectiveness data are not yet available from follow-up tenofovir trials being conducted in South Africa, Uganda, and Zimbabwe (1 trial) and multiple sites in South Africa (1 trial). We found no evidence of a significant effect for nonoxynol-9 (5 trials), cellulose sulphate (2 trials), SAVVY (2 trials), Carraguard (1 trial), PRO 2000 (2 trials), and BufferGel (1 trial) microbicides. The pooled RR for the effect of current experimental vaginal microbicides on HIV acquisition in women was 0.97, 95%CI 0.87 to 1.08. Although study results were homogeneous across the different drug classes (heterogeneity P = 0.17, I^2^ = 27%), the overall intervention effect was not statistically significant. Nonoxynol-9 significantly increased the risk of having adverse genital lesions but no other microbicide led to significant increases in adverse events.

**Conclusions:**

There is not enough evidence at present to recommend vaginal microbicides for HIV prevention. Further high-quality research is needed to confirm the beneficial effects of tenofovir as well as continue the development and testing of new microbicides.

## Background

Antiretroviral drugs are among the greatest medical breakthroughs of the last three decades. However, they have limitations which include (but are not limited to) the emergence of multi-drug-resistant HIV virus strains, toxicity, difficult treatment regimens, and inadequate pharmacology, bioavailability and tissue distribution [1-3]. Therefore, prevention of new HIV infections remains the backbone of efforts to control the HIV pandemic [1,4,5]. The male condom protects against acquisition and transmission of HIV, but its use requires agreement by both sexual partners. Recently, early initiation of antiretroviral therapy was shown to be effective in reducing the risk of sexual transmission of HIV [6]. In addition medical male circumcision reduces the risk of HIV acquisition in men [7,8], but evidence is lacking on whether male circumcision confers protection for women; who currently account for more than half of the 34 million people estimated to be living with HIV worldwide [1]. The need for female-controlled HIV prevention strategies has been recognised, and the current strategies being researched include vaginal microbicides [9]. Microbicides are compounds that when inserted vaginally would (at least in theory) act to prevent acquisition and or transmission of HIV during sexual intercourse. Most of the current microbicide research is undertaken in sub-Saharan Africa and Southeast Asia, but an effective microbicide will undoubtedly be used worldwide to prevent the sexual acquisition and or transmission of HIV. It has been estimated that even a partially effective microbicide could prevent millions of new HIV infections each year throughout the world [10]. In the absence of an effective prophylactic HIV vaccine, the development of a safe and effective microbicide is critical. This review aimed to determine the effectiveness of vaginal microbicides in preventing sexual acquisition of HIV infection by women.

## Methods

We undertook this systematic review according to standard Cochrane methods [11]. We conducted a comprehensive search of electronic databases for relevant randomised controlled trials and systematic reviews published by September 2012. The databases searched were the Cochrane Central Register of Controlled Trials (CENTRAL), Medline, Embase, Web of Science, NLM Gateway, AIDS Education Global Information System, ClinicalTrials.gov, the World Health Organization (WHO) International Clinical Trials Registry Platform, Cochrane Database of Systematic Reviews (CDSR), and York Database of Abstracts of Reviews of Effectiveness (DARE). We supplemented the search by screening bibliographies of identified articles and proceedings of international AIDS conferences, and contacting relevant experts at WHO and the Joint United Nations Programme on HIV/AIDS (UNAIDS). We searched for all relevant studies regardless of language or publication status.

**Figure 1:**
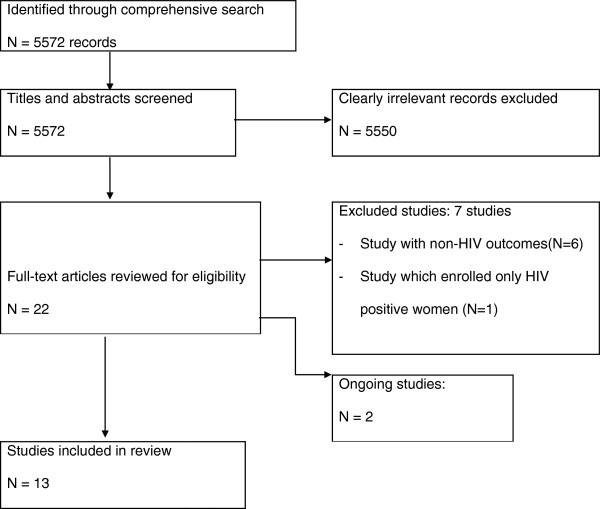
Flow chart of identification and selection of studies for inclusion.

Two authors (Jael Obiero and Charles Wiysonge) independently assessed study eligibility, risk of bias in included studies, and extracted data; with disagreements resolved by discussion and consensus. Eligible studies were randomised controlled trials in which sexually active HIV-negative women from any setting were randomly allocated to a vaginal microbicide compared to a placebo or no intervention. All included studies were approved by relevant institutional ethical review boards in specific countries and all participants provided written informed consent. We have summarised the search and selection process for this review in Figure 1.

Our outcome of interest was new HIV infection; determined using internationally accepted diagnostic criteria.

We used the Cochrane Collaboration’s Review Manager 5.1 ( http://ims.cochrane.org/RevMan) for statistical analyses. We expressed each study result as a risk ratio (RR) with its 95% confidence intervals (CI) and conducted meta-analysis by analysing trial participants in groups to which they were randomised, regardless of which or how much treatment they actually received. Despite the excellent mathematical properties of the odds ratio (OR), we did not choose it to measure study results in our analyses because the OR and RR differ considerably when control group risks are high (as was the case in this review) and effects are large. A misinterpretation of the OR in such a situation would lead to an overestimation of the intervention effect. There exist published examples of situations where authors have misinterpreted the OR from meta-analyses as if they were RR, leading to overestimation of intervention effects [11].

In meta-analyses, it is important to measure the degree to which the results of studies are consistent. Inconsistency in study results, known as statistical heterogeneity, is a consequence of clinical and or methodological differences among studies. Statistical heterogeneity manifests itself in the observed treatment effects being more different from each other than one would expect due to random error (or chance) alone. We used the Cochran’s Q test to assess statistical heterogeneity between study results, defining statistical significance at the 10% alpha level [11,12]. Q is a chi-squared statistic with “degrees of freedom” equals to “number of studies” minus 1. The statistic is calculated as the weighted sum of the squared differences between individual study effects and the pooled effect across the included studies, with the weights defined by the model of meta-analysis used i.e. fixed effect or random-effects. Q assesses whether observed differences in results are compatible with chance alone. A low P value (or a large chi-squared statistic relative to its degrees of freedom) provides evidence of significant statistical heterogeneity of intervention effects [11,12]. In addition, we used the Higgins’ I-squared test to quantify inconsistency in the effects of microbicides across the studies included in the meta-analysis [13]. The I-squared test statistic describes the percentage of the variability in effect estimates that is due to true differences between studies rather than chance.

There was no significant statistical heterogeneity between trial results (X^2^ = 17.72 [13 degrees of freedom], P = 0.17; I^2^ = 27%), and we combined the results using fixed effect meta-analysis [11,12]. However we also conducted a random-effects meta-analysis [14], in order to document the robustness of our findings to the method of meta-analysis [11]. The pooled effect from the fixed-effect method is considered to be a typical intervention effect from the studies included in the meta-analysis; implying that observed differences among study results are caused exclusively by chance. Random-effects meta-analysis, on the other hand, assumes that estimated study effects are not identical; but differ at random, following a normal distribution [14]. In addition, we sub-grouped the trials by type of microbicide under investigation.

## Results

Of 22 potentially eligible studies, 13 met our inclusion criteria [15-27] and two are ongoing [28,29]. The remaining seven studies were excluded; six of them enrolled HIV-negative women but did not report HIV incidence [30-35] and the seventh one enrolled only HIV positive women [36]. The seven excluded trials were set up to assess the effects of vaginal microbicides on the incidence of non-HIV sexually transmitted infections [30-36].

The 13 included randomised controlled trials, involving seven microbicides, had either been conducted to term (three Nonoxynol-9, one BufferGel and 0.5% PRO 2000, one Carraguard, and one tenofovir trial) or stopped early due to safety concerns (one Nonoxynol-9 and two cellulose sulphate trials) or insufficient rate of HIV infection and low likelihood of showing a protective effect (one Nonoxynol-9, one 2% PRO 2000, and two SAVVY trials) (Table 1). The studies enrolled 35,905 sexually active HIV negative women between 1996 and 2011; in Kenya [15,19], Cameroon [16,17], Cote d’Ivoire [18], Benin [18,24], Ghana [23], Malawi [26], Nigeria [20,21], South Africa [17,22,24-27], Tanzania [22], Uganda [22], Zambia [22,26], Zimbabwe [26], Thailand [18], India [24], and the United States of America [26].

**Table 1:** A summary of characteristics of included studies

**Author**	**Method**	**Participants and settings**	**Interventions/control**
Kreiss 1992 [15]	Unblinded RCT	138 female sex workers Nairobi, Kenya	1000 mg N-9 sponge/placebo
Richardson 2001 [19]	Double blind RCT	278 female sex workers, Mombasa, Kenya	52.5 mg N-9 gel/placebo
Roddy 1998 [17]	Double blind RCT	1292 female sex workers, Cameroon	70 mg N-9 film/placebo
Roddy 2002 [16]	Unblinded RCT	1251 women (non sex workers) from clinics or pharmacy, Cameroon	100 mg N-9 gel/No intervention
Van Damme 2002 [18]	Triple blind RCT	892 women sex workers from Benin, Cote d’Ivoire, South Africa, Thailand	52.5 mg N-9/placebo
Halpern 2008 [21]	Double blind RCT	1644 women from bars, market, other common gathering areas, Lagos and Port Harcourt, Nigeria	6% CS gel/placebo
Van Damme 2008 [24]	Double blind RCT	1428 women, South Africa, Uganda, Benin and India (Chennai, Bangalore)	6% CS gel/placebo
Felblum 2008 [20]	Double blind RCT	2153 women from local market areas, bars hostels, military barracks and colleges, Lagos and Ibadan, Nigeria	1.0% C31G (SAVVY) gel/placebo
Peterson 2007 [23]	Double blind RCT	2142 women from high HIV transmission areas including markets, bars, hotels, Accra and Kumasi, Ghana	1% C31G (SAVVY) gel/placebo
Skoler-Karpoff 2008 [27]	Double blind RCT	6,202 women from local health clinics, malls, churches, taxi ranks, other community venues, University of Cape Town, University of Limpopo-Medunsa campus Ga-Rankuwa and Medical Research Council, Durban, South Africa	Carraguard gel/placebo
Abdul Karim 2011 [26]	RCT Double blind	3101 women in Malawi, South Africa, Zambia , Zimbawe, USA	BufferGel/ 0.5% PRO2000/placebo
McCormack 2010 [22]	RCT Double blind	6,651 women, three sites in South Africa; Mwanza, Tanzania; Entebbe, Uganda; Mazambuka, Zambia	0.5% PRO2000 gel/placebo
Abdul Karim 2010 [25]	RCT Double blind	1085 at urban and rural clinic, Kwa-Zulu Natal, South Africa	1% tenofovir gel/placebo

*CS*, cellulose sulphate; *N-9*, nonoxynol-9; RCT, randomised controlled trials; *USA*, United States of America.

A small proof-of-concept randomised controlled trial found that tenofovir (a nucleotide reverse transcriptase inhibitor) significantly reduces the risk of HIV acquisition (889 women: RR 0.63, 95% CI 0.43 to 0.93). Despite the small sample size, the results were statistically significant (P = 0.02) [25]. Effectiveness data are not yet available from the second tenofovir trial that enrolled 5000 women (in South Africa, Uganda, and Zimbabwe) and was stopped early due to low likelihood of showing a protective effect [28]. Two of the five arms in this trial were designed to test the effectiveness of the tenofovir gel and enrolled 2000 women [28]. The third tenofovir trial started in October 2011, with data collection expected to end in May 2014 [29]. The latter is planned to assess the safety and effectiveness of 1% tenofovir gel among approximately 2900 sexually active women at high risk for sexual acquisition of HIV in South Africa.

**Figure 2:**
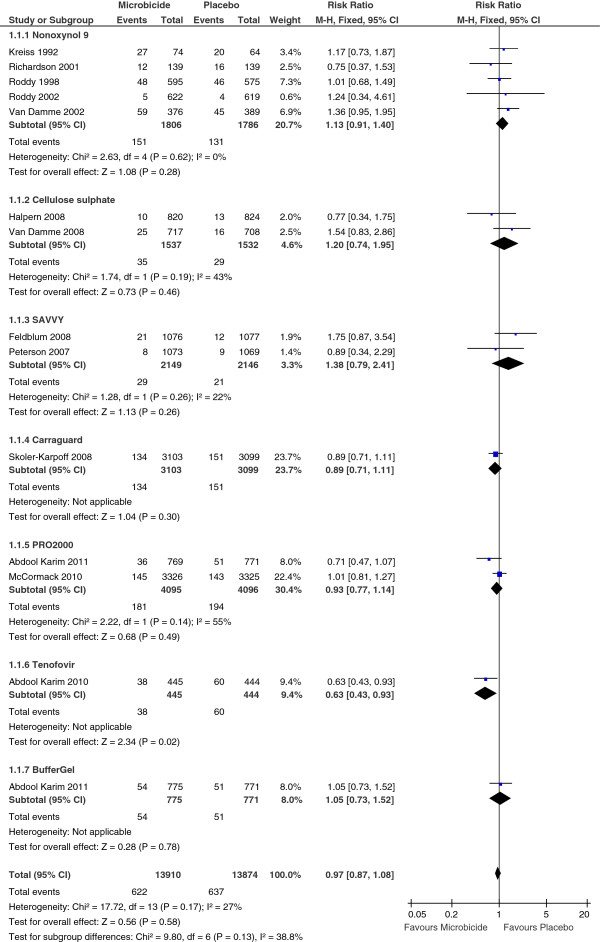
**Effectiveness of experimental vaginal microbicides compared to placebo or no intervention for prevention of HIV acquisition in women.** n = number infected: N = total number of participants.

We found no evidence of a significant effect on HIV acquisition for nonoxynol-9 (5 trials, N = 3592; RR 1.13, 95% CI 0.91 to 1.14; [15-19]), cellulose sulphate (2 trials, N = 3,069: RR 1.20, 95% CI 0.74 to 1.95; [21,24]), SAVVY (2 trials, N = 4,295: RR 1.38, 95% CI 0.79 to 2.41; [20,23]), Carraguard (1 trial, N = 6,202: RR 0.89, 95% CI 0.71 to 1.11; [27]), PRO 2000 (2 trials, N = 12,486: RR 0.93, 95% CI 0.77 to 1.14, [22,26]), and BufferGel (1 trial, N = 1,546: RR 1.05, 95% CI 0.73 to 1.52; [26]) (Figure 2).

The pooled RR for the 13 trials was 0.97 (95% CI 0.87 to 1.08) when we used fixed effect meta-analysis, and 0.98 (95% CI 0.86 to 1.12) when we used the random-effects method. Although the confidence intervals were wider with the random-effects method, the findings of our review are not sensitive to the method of meta-analysis.

The most common adverse event reported by trials was genital lesion (laceration, abrasion, ulceration, irritation) with the highest risk occurring in the nonoxynol-9 trials (5 trials, N = 3592). In the other trials this event was reported to be similar in both the microbicide and placebo arms.

**Figure 3:**
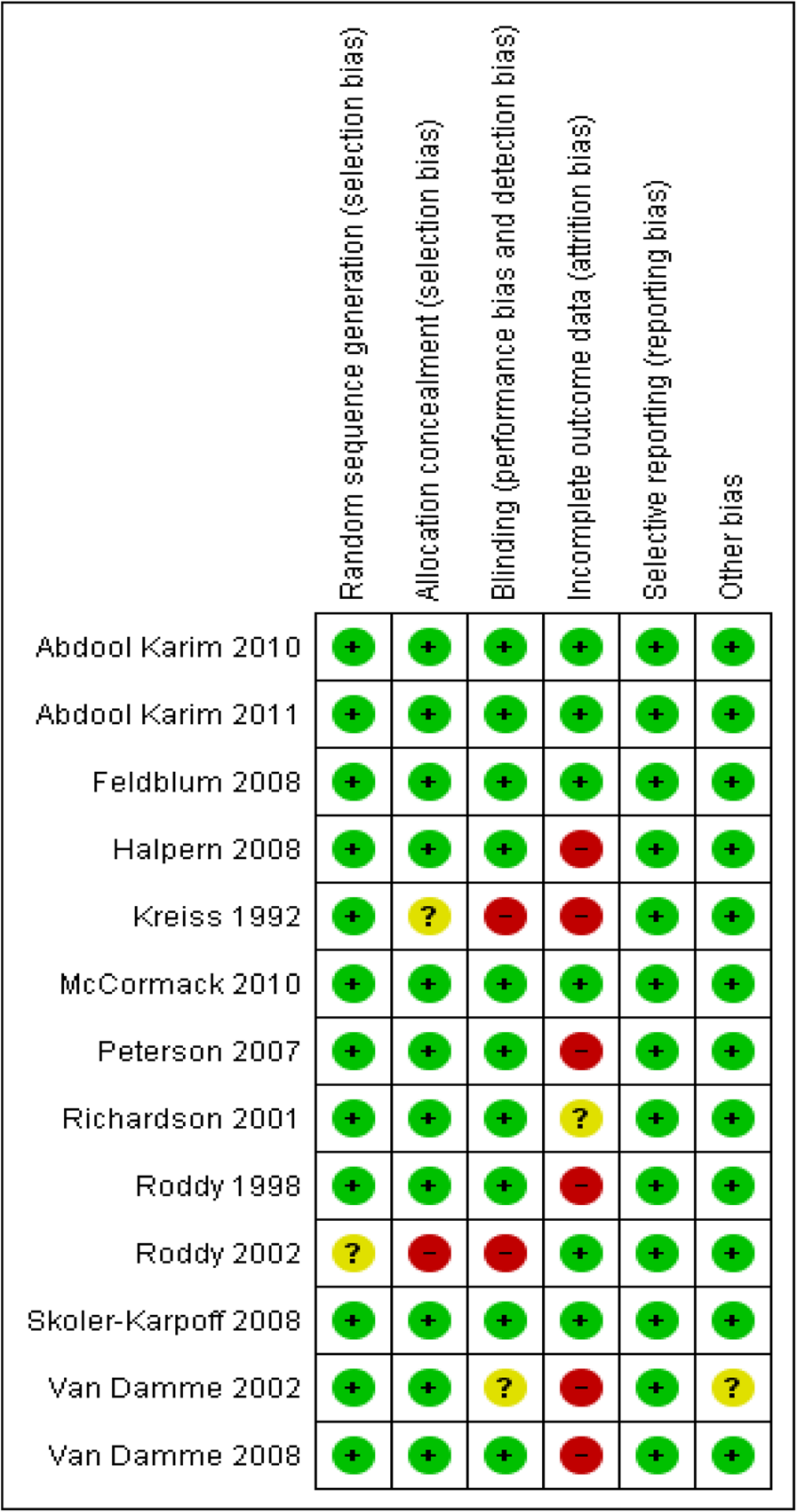
The risk of bias in currently available randomised controlled trials that have assessed the effect of vaginal microbicides for prevention of HIV acquisition in women.

The randomisation sequence was adequately generated and the allocation was adequately concealed in all 13 studies. We therefore think there was no selection bias in any of the included studies. In two studies [15,16] blinding was not done since an appropriate placebo could not be manufactured; hence high risk of bias. Seven trials [15,19-24] were stopped early due to data-dependent processes. Due to the early discontinuation, four of the studies had a very high attrition rate: 10% in a cellulose sulphate trial [24], 16% in a SAVVY trial [23], 17% in a nonoxynol-9 trial [15], and 30% in another cellulose sulphate trial [21]. In addition, loss to follow up was high in three nonoxynol-9 trials conducted to term i.e. 12% in the trial by Rody and colleagues among non-sex workers in Cameroon [16], 20% in the trial by Rody and co-workers among female sex workers in Cameroon [17], and 22.6% in a trial by Van Damme and co-workers among female sex workers in Benin, Cote d’Ivoire, South Africa and Thailand [18]. We have rated the risk of bias due to incomplete outcome data for each of these studies as high. The proportion of participants lost to follow up was unclear in one nonoxynol-9 study [19]. The remaining studies had losses to follow-up ranging from 3% to 7% [20,22,25-27]. We have summarised the risk of bias in included trials in Figure 3.

## Discussion

To date, 13 randomised controlled trials involving seven different vaginal microbicides (namely: nonoxynol-9, SAVVY, cellulose sulphate, carraguard, PRO 2000, BufferGel and tenofovir) have either been conducted to term or stopped early at the recommendation of the respective data and safety monitoring boards. Overall, the included studies which enrolled a total of 35,905 HIV-negative heterosexual women show no evidence of an effect of vaginal microbicides on the risk of HIV acquisition. The pooled risk ratio was 0.97 (95% CI 0.87 to 1.08) for fixed-effect and 0.98 (95% CI 0.86 to 1.12) for random-effects meta-analysis (I^2^ = 27%). However, one small study that enrolled 889 women in South Africa suggests that a vaginal microbicide containing tenofovir (an antiretroviral drug belonging to the class of nucleotide reverse transcriptase inhibitors) may be effective in reducing the risk of HIV acquisition in heterosexual women. The relatively small sample size and the small number of study sites may restrict the broad generalisability of the finding that tenofovir reduces the risk of HIV acquisition in heterosexual women.

The main strengths of our systematic review are the comprehensive search for published and unpublished studies in multiple databases and contact with relevant organisations and experts, quantitative synthesis of all currently available data, and sensitivity analyses to investigate the sensitivity of our findings to the method of meta-analysis. In general, the Q statistic that we used to assess for heterogeneity in trial results has low power when studies included in a meta-analysis have small sample size or are few in number. This implies that, although a statistically significant result indicates substantial inconsistency in study effects, a non-significant result does not necessarily mean that there is no heterogeneity of effects [11,13]. Therefore to make our findings more secure, despite the finding of no significant statistical heterogeneity of effects, we conducted and reported both fixed- effect and random-effects meta-analyses [12,14]. In addition, it has been argued that statistical heterogeneity is inevitable in a meta-analysis; given that there will always be clinical and or methodological differences (however small) between studies included in the meta-analysis [13]. We therefore used the Higgins’ I-squared statistic to quantify the heterogeneity of intervention effects across included studies, even though the Q statistic did not detect significant statistical heterogeneity. The low I-squared value (i.e. 27%) reassured us that heterogeneity did not have a significant impact on the pooled effect estimate [11,13].

To the best of our knowledge, although other microbicide reviews have been conducted [37-42], our review is the most comprehensive synthesis of existing evidence on experimental vaginal microbicides for prevention of HIV acquisition in women. There are substantive differences between the current systematic review and the Cochrane review of topical microbicides for prevention of sexually transmitted infections [42]. Firstly, we have included all trials of experimental vaginal microbidides tested to date; but the Cochrane review excluded trials that assessed the effects of nonoxynol-9. Secondly, the current review assessed the effects of experimental microbicides on HIV acquisition while the Cochrane review focused on sexually transmitted infections. Thirdly, the current review included only studies that enrolled heterosexual women who were HIV-negative at enrolment whereas the Cochrane review searched for studies involving heterosexual women or men who have sex with men who had no evidence of sexually transmitted infections at enrolment. Finally, the current review includes a quantitative synthesis of all currently available effectiveness data from experimental vaginal microbicides; but the Cochrane review conducted a narrative synthesis of effects across the different classes of non-nonoxynol-9 microbicides.

## Conclusions

At present, limited evidence suggests that vaginal microbicides containing tenofovir - a nucleotide reverse transcriptase inhibitor - may reduce HIV acquisition in heterosexual women; but other types of vaginal microbicides have not shown evidence of an effect. Therefore, there is not enough evidence to recommend vaginal microbicides for HIV prevention at present. Further randomised controlled trials are needed to confirm the beneficial effects of the tenofovir gel. In addition, further research should continue on the development and testing of new microbicides.

## Competing interests

The authors have declared that they have no competing interests.

## Authors’ contributions

JO and CSW conceived the review, selected studies, extracted and analysed data, and wrote the first draft of the manuscript. PGM and GDH contributed to the interpretation of the results and critical revisions of the manuscript. All authors read and approved the final manuscript.

## Pre-publication history

The pre-publication history for this paper can be accessed here:

http://www.biomedcentral.com/1471-2334/12/289/prepub
